# Human Perception of Fear in Dogs Varies According to Experience with Dogs

**DOI:** 10.1371/journal.pone.0051775

**Published:** 2012-12-19

**Authors:** Michele Wan, Niall Bolger, Frances A. Champagne

**Affiliations:** Department of Psychology, Columbia University, New York, New York, United States of America; Université de Strasbourg, France

## Abstract

To investigate the role of experience in humans’ perception of emotion using canine visual signals, we asked adults with various levels of dog experience to interpret the emotions of dogs displayed in videos. The video stimuli had been pre-categorized by an expert panel of dog behavior professionals as showing examples of happy or fearful dog behavior. In a sample of 2,163 participants, the level of dog experience strongly predicted identification of fearful, but not of happy, emotional examples. The probability of selecting the “fearful” category to describe fearful examples increased with experience and ranged from.30 among those who had never lived with a dog to greater than.70 among dog professionals. In contrast, the probability of selecting the “happy” category to describe happy emotional examples varied little by experience, ranging from.90 to.93. In addition, the number of physical features of the dog that participants reported using for emotional interpretations increased with experience, and in particular, more-experienced respondents were more likely to attend to the ears. Lastly, more-experienced respondents provided lower difficulty and higher accuracy self-ratings than less-experienced respondents when interpreting both happy and fearful emotional examples. The human perception of emotion in other humans has previously been shown to be sensitive to individual differences in social experience, and the results of the current study extend the notion of experience-dependent processes from the intraspecific to the interspecific domain.

## Introduction

The ability to perceive and recognize emotion in others is a fundamental human social cognitive skill, facilitating interpersonal interaction, social learning, and empathic behavior [1], [2]. These abilities vary according to experience. Neglected children, for example, experience difficulty in discriminating among facial expressions due to a deficit in socioemotional information during development [3]. Abused children are over-exposed to anger and are consequently hyper-responsive to angry expressions, tending to categorize more expressions as angry, yet displaying typical categorization of fearful, happy, and sad facial expressions [3]–[6]. Even the level of cross-cultural experience, as measured by geographical distance and amount of telephone communication, is associated with accuracy in facial expression recognition between cultures [7], [8].

Several recent studies have investigated the role of experience on interpretations of emotion in dogs [9]–[11]. Due to their unique relationship with humans, dogs are a prime candidate for such investigations. Interspecific experience varies widely, allowing for a great range of experiential comparisons, perhaps greater than the range provided by intraspecific experience. For example, perceptions of emotion in dogs can be compared between individuals who have never interacted regularly with dogs and those who have worked professionally with many dogs. Investigations of interspecific emotion perception may present a promising, new strategy for understanding the role of experience in the development of emotion perception and other social cognitive abilities.

Recent research findings suggest that experience with dogs influences the neural processing of dog behavior. An fMRI study found that the brain activity of dog experts differed from that of non-experts as they viewed dog images and suggested that experts’ brains differentiate dog body postures in a similar manner as they distinguish human body postures [12]. In addition, eye gaze patterns on dog images were found to vary with experience. Since experience with dogs appears to influence both visual and neural activity, it is reasonable to suggest that social cognitive abilities, such as emotion perception, also differ according to experience with dogs.

Several studies have examined the role of experience in the perception of emotion in dogs by asking participants with disparate levels of dog experience to interpret auditory or visual dog signals [9]–[11]. The results suggest that the perception of emotion using auditory cues (dog barks) does not vary greatly by experience. For example, Pongrácz et al. [10] asked listeners with various levels of dog experience to identify the context and emotional content of played-back dog barks. Owners of the breed whose barks were played, owners of other breeds, and non-owners did not differ significantly from each other in categorizing barks by situation, and differences by experience in emotionality ratings were minor. Similarly, Molnár et al. [9] asked individuals with varying visual experience to interpret barks. They compared congenitally blind individuals, blind individuals with previous visual experience, and sighted individuals. Individuals with greater visual experience would have had increased opportunity for visual processing of the situations in which various barks occur, as well as observation of the visual signals that accompany barks. As in the previous study, there were no significant differences among the experience groups in categorizing barks by situation, and in most cases, ratings of the barks’ emotionality also did not vary by experience.

In contrast to dog vocalizations, there is some evidence for the role of experience in the perception of emotion using canine visual signals. In studies of pre-adolescent children, there appears to be a strong effect of age in the interpretation of visual signals [13]–[15]. For example, 4-year-old children are significantly more likely than 6-year-old children to misidentify aggressive dog faces as happy. However, it is unclear whether these age-dependent effects are due to increased lifetime experience with dogs or simply the maturation of emotion processing systems. Among adults, there is limited evidence for the role of experience in interpretations of canine visual signals. Tami and Gallagher [11] asked adults with varying dog experience, namely veterinarians, dog trainers, dog owners, and non-owners, to interpret videos of dog-dog interactions and found few differences as a function of experience. However, high variability in emotional interpretations were evident, even among dog professionals, suggesting that the ritualized nature of dog-dog interactions may contribute to idiosyncratic interpretations, which could obscure experience-related differences. Thus, further work on the role of experience in interpretations of canine visual signals outside the context of dog-dog interactions is needed.

To investigate the role of experience in humans’ perception of emotion using canine visual signals, we asked adults with various levels of dog experience to interpret the emotions of dogs displayed in videos, in everyday scenarios other than dog-dog interactions. Following the approach commonly used in human intraspecific emotion perception research, we focus here on participants’ categorical perception of emotion. The most common emotion categories included in intraspecific studies are anger, fear, happiness, sadness, disgust, and surprise – often called primary emotions [7], [16]. The first four of these emotions were included as response choices in the current study, because neurobehavioral evidence supports the existence of similar affective states in animals [17]. In addition, more than 60% of dog owners perceive these emotions in their dogs [18]. The videos were embedded in an online questionnaire, in which participants categorized each dog’s emotional state and reported the features of the dog that informed them about the dog’s emotional state. Respondents rated the difficulty that they experienced in interpreting each dog’s emotions, as well as their own perceived accuracy. We hypothesized that the level of experience with dogs would be associated with all measures, supporting intraspecific findings that emotion perception processes are shaped by experience.

## Results

### Sample Characteristics

A total of 2,163 participants completed the questionnaire and were included in the analyses that follow. Of these participants, 82% were female, and 18% were male. The age of the participants ranged from 18 to 84 with a mean age of 41.44 (*SD = *15.06). Among respondents, 91% resided in the United States, while 9% resided in other countries, such as Australia, Canada, and the United Kingdom.

Seven percent of the respondents had never owned a dog and reported having no experience with dogs or only occasional experience (Low-Experience group, *n* = 152). Sixty-eight percent reported having a dog at some point in their lives (Owners group, *n* = 1,462). Fourteen percent reported that they had worked professionally with dogs from one to nine years (Prof<10 group, *n* = 307), while 11% had worked professionally with dogs for ten or more years (Prof10+ group, *n* = 242). Only six individuals from the professional groups reported that they had never owned a dog. Among the professionals, 70% were dog behavior professionals, such as trainers and applied animal behaviorists, while 30% worked in fields not primarily associated with behavior, such as dog grooming, dog sitting (caring for pet dogs while their owners are away), and non-behavioral veterinary care.

### Emotion Categorizations

Video stimuli had been pre-categorized by an expert panel of dog behavior professionals as showing examples of happy or fearful dog behavior (see *Methods*). Interpretations of these videos by the various experience groups in the full sample are the focus of the analyses below. Specifically, we examined whether respondents’ emotion categorizations matched experts’ emotion categorizations. A raw breakdown of categorizations for each experience group is displayed in Tables 1 and 2. As described in the *Statistical Analyses*, all reported models in the *Results* account for the effects of participant’s sex and age, as well as effects of individual videos.

**Table 1:** Emotion Categorizations of Happy Emotional Examples.

Experience Group	Happy	Fearful	Angry	Sad	Neutral
Low-Exp	89.9%	3.6%	0.3%	0.8%	5.4%
Owners	92.5%	1.2%	0.3%	0.1%	6.0%
Prof<10	91.1%	2.9%	0.1%	0.1%	5.7%
Prof10+	89.8%	1.9%	0.2%	0.2%	7.9%

Percentages indicate number of selections of each category out of total number of responses by each experience group.

**Table 2:** Emotion Categorizations of Fearful Emotional Examples.

Experience Group	Happy	Fearful	Angry	Sad	Neutral
Low-Exp	16.6%	34.7%	13.1%	7.8%	27.8%
Owners	9.9%	59.9%	2.7%	4.1%	23.4%
Prof<10	5.5%	71.9%	2.8%	3.1%	16.6%
Prof10+	3.9%	72.0%	1.6%	3.3%	19.1%

Percentages indicate number of selections of each category out of total number of responses by each experience group.

**Figure 1:**
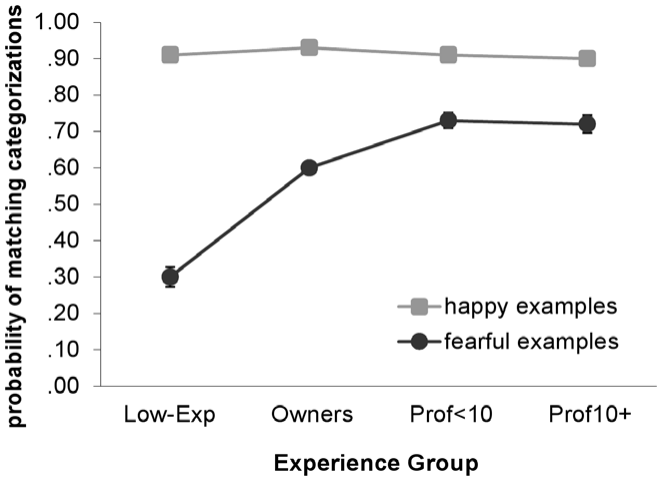
Emotion categorizations according to experience with dogs. Probability of “happy” categorizations of happy emotional examples and “fearful” categorizations of fearful emotional examples by experience group. Error bars represent standard errors of the means. Model-fitted values account for effects of participant’s sex, participant’s age, and individual videos. Sig. pairwise comparisons (Sidak-corrected): Fearful examples: Low-Exp< Own <Prof<10 = Prof10+.

Experience predicted identification of fearful, but not happy, behavior. In other words, the probability of selecting the “happy” category to describe happy emotional examples did not vary by experience, *Wald X^2^*(3, *N* = 2163) = 7.18, *P* = .07; range:.90–.93. In contrast, experience was strongly associated with selection of the “fearful” category for fearful emotional examples, *Wald X^2^*(3, *N* = 2163) = 152.67, *P*<.001 (Figure 1). For these videos, the likelihood of selecting the “fearful” category increased dramatically with the level of dog experience and ranged from.30 in the Low-Exp group to greater than.70 in both professional groups.

In addition, the results were maintained when participants’ ratings of the likeliness that dogs experience fear or happiness were also controlled in the analyses, *Wald X^2^*(3, *N* = 2163) = 151.10, *P*<.001 and *Wald X^2^*(3, *N* = 2163) = 7.16, *P* = .07. Furthermore, when the analyses focused on hands-on experience by eliminating individuals who reported that they had learned about dog body language by reading a book or article, watching a video, attending a lecture, or receiving an explanation from a behavior professional, experience remained a significant predictor of identification of fearful, but not happy, emotional examples, *Wald X^2^*(2, *N* = 630) = 38.32, *P*<.001 and *Wald X^2^*(2, *N* = 630) = .52, *P* = .77.

### Observational Focus

**Figure 2:**
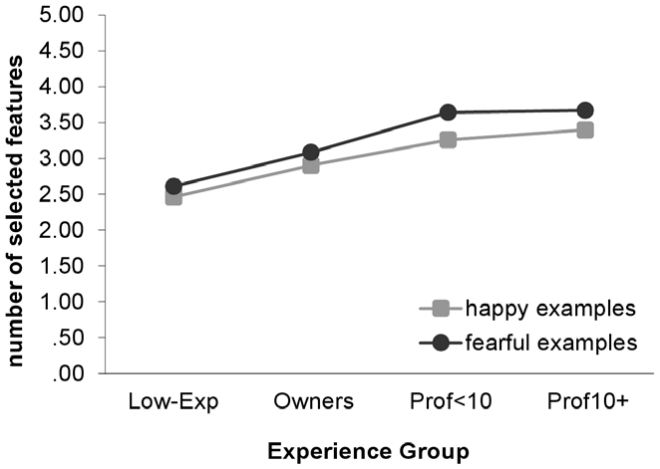
Number of physical features selected **according to experience with dogs.** Number of features reported by participants as emotionally informative; by experience group. Response choices consisted of “eyes,” “ears,” “mouth/tongue,” “legs/paws,” and “tail.” Error bars represent standard errors of the means. Model-fitted values account for effects of participant’s sex, participant’s age, and individual videos. Sig. pairwise comparisons (Sidak-corrected): Happy: Low-Exp< Own <Prof<10 = Prof10+. Fearful: Low-Exp< Own <Prof<10 = Prof10+.

For each dog, participants could also indicate which major physical features of the dog informed them about the dog’s emotional state (eyes, ears, mouth/tongue, legs/paws, tail). Experience was a significant predictor of the number of features that participants selected for both happy and fearful emotional examples, *Wald X^2^*(3, *N* = 2163) = 153.77, *P*<.001 and *Wald X^2^*(3, *N* = 1921) = 158.55, *P*<.001. The number of features increased with experience, and all groups differed significantly, except for the two professional groups (Figure 2). Out of a total of five listed features, the Prof10+ group (*M* = 3.40 for happy and 3.67 for fearful) selected approximately one more feature than the Low-Exp group (*M* = 2.46 for happy and 2.61 for fearful) when viewing examples of both emotions. On average, more features were selected for fearful (*M* = 3.22) than happy examples (*M* = 2.98).

**Figure 3:**
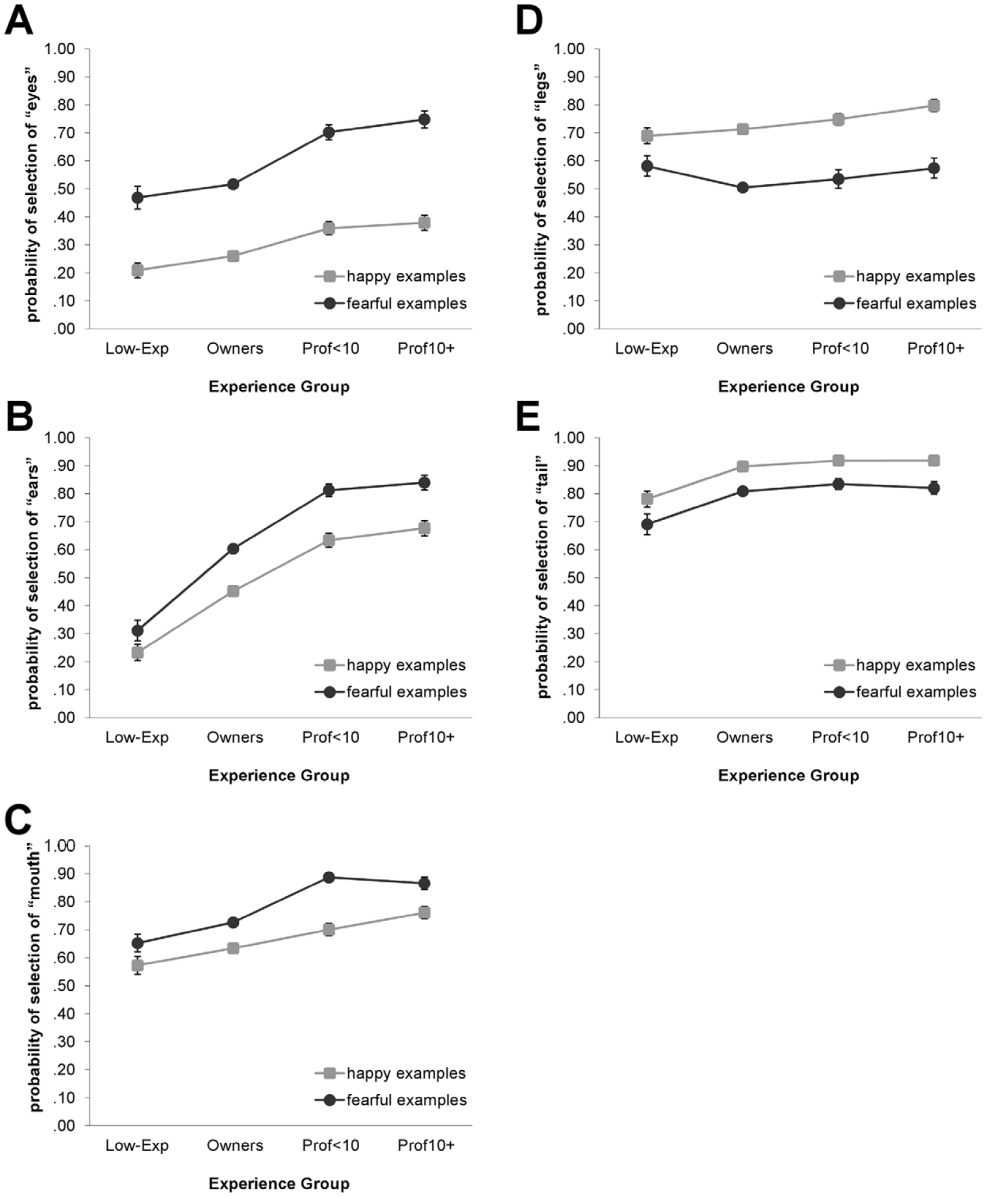
Probability of reporting particular features as emotionally informative, according to experience with dogs. A) eyes, B) ears, C) mouth/tongue, D) legs/paws, and E) tail. Error bars represent standard errors of the means. Model-fitted values account for effects of participant’s sex, participant’s age, and individual videos. Sig. pairwise comparisons (Sidak-corrected): Eyes - Happy: Low-Exp = Own <Prof<10 = Prof10+; Fearful: Low-Exp = Own <Prof<10 = Prof10+. Ears - Happy: Low-Exp< Own <Prof<10 = Prof10+; Fearful: Low-Exp< Own <Prof<10 = Prof10+. Mouth - Happy: Low-Exp = Own <Prof<10 = Prof10+; Fearful: Low-Exp = Own <Prof<10 = Prof10+. Legs - Happy: Low-Exp = Own <Prof10+. Tail - Happy: Low-Exp< Own = Prof<10 = Prof10+; Fearful: Low-Exp< Own = Prof<10 = Prof10+.

In addition, as displayed in Figure 3, facial features (eyes, ears, mouth/tongue) were more likely to be reported as informative for fearful than happy videos, while the opposite was found for bodily features (legs/paws, tail). The probability of selection of each feature also varied by experience with generally greater differences observed in the selection of facial than bodily features. The largest differences by experience occurred for the “ears” category, for which the probability of selection increased significantly from the Low-Exp to the professional groups [happy: *Wald X^2^*(3, *N* = 2163) = 145.72, *P*<.001; fearful: *Wald X^2^*(3, *N* = 1921) = 170.24, *P*<.001]. Non-professionals were also less likely than professionals to report that the eyes and mouth were emotionally informative, though the differences were not as large as for the ears [Happy: eyes, *Wald X^2^*(3, *N* = 2163) = 42.20, *P*<.001; mouth, *Wald X^2^*(3, *N* = 2163) = 38.61, *P*<.001. Fearful: eyes, *Wald X^2^*(3, *N* = 1921) = 73.85, *P*<.001; mouth, *Wald X^2^*(3, *N* = 1921) = 115.84, *P*<.001]. Selection of the “legs” category did not vary by experience for fearful emotional examples, but non-professionals were less likely than the Prof10+ group to select this category when viewing happy examples [happy: *Wald X^2^*(3, *N* = 2163) = 15.03, *P* = .002; fearful: *Wald X^2^*(3, *N* = 1921) = 7.16, *P* = .07]. Selection of the “tail” category also varied with experience; the Low-Exp group was less likely than all other groups to report that this feature was informative in both happy and fearful videos [happy: *Wald X^2^*(3, *N* = 2163) = 33.35, *P*<.001; fearful: *Wald X^2^*(3, *N* = 1921) = 16.13, *P* = .001].

### Difficulty and Accuracy

**Figure 4:**
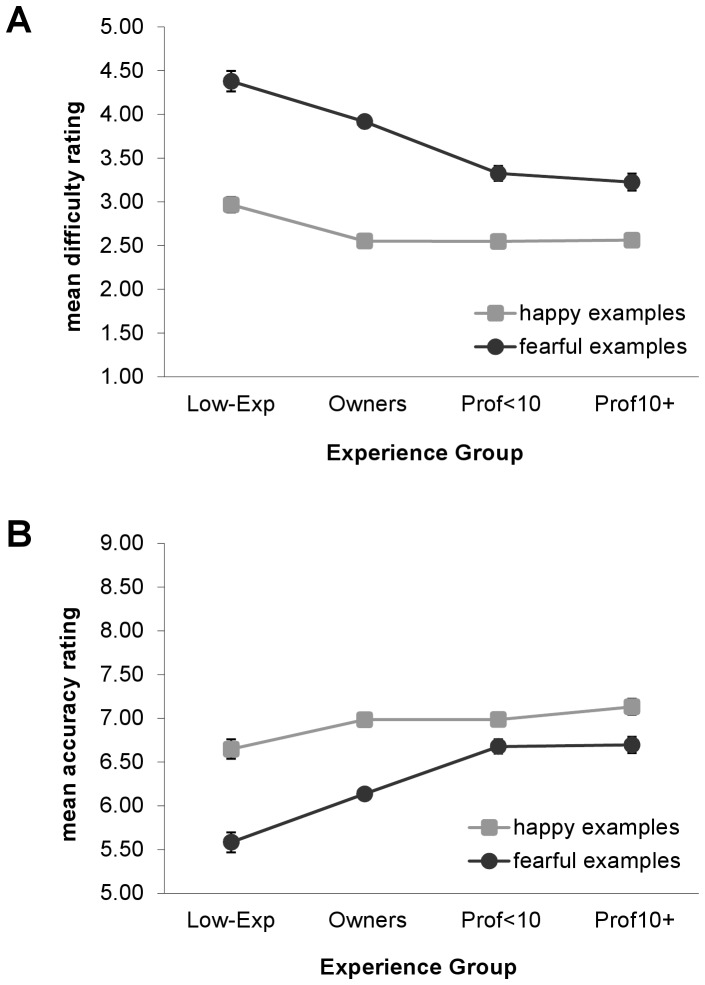
Self-reported difficulty and accuracy ratings according to experience with dogs. A) Difficulty ratings and B) accuracy ratings for interpretations of happy and fearful examples by experience group. Difficulty ratings: 1 = very easy, 9 = very difficult. Accuracy ratings: 1 = very inaccurate; 9 = very accurate. Error bars represent standard errors of the means. Model-fitted values account for effects of participant’s sex, participant’s age, and individual videos. Sig. pairwise comparisons (Sidak-corrected): Difficulty - Happy: All Others<Low-Exp; Fearful: Prof10+ = Prof<10< Own<Low-Exp. Accuracy - Happy: Low-Exp< Own = Prof10+; Fearful: Low-Exp< Own <Prof<10 = Prof10+.

Respondents from all experience groups rated happy emotional examples as easier to interpret than fearful examples and perceived their interpretations to be more accurate for happy than fearful examples (Figure 4). However, less-experienced respondents, particularly the Low-Experience group, reported greater difficulty and lower accuracy than more-experienced respondents when interpreting both happy and fearful examples [difficulty: happy, *F*(3, 4918) = 7.00, *P*<.001, fearful, *F*(3, 4319) = 36.03, *P*<.001; accuracy: happy, *F*(3, 5388) = 3.82, *P* = .01, fearful, *F*(3, 4319) = 33.59, *P*<.001]. Furthermore, differences by experience in difficulty and accuracy ratings were greater for interpretations of fearful than happy examples. For instance, mean difficulty ratings for happy examples differed between the Low-Exp and Prof10+ groups by 0.41 points on a nine-point scale, whereas the difference was more than twice as large (1.16 points) for fearful examples. Lastly, respondents who selected the “happy” category for happy examples or the “fearful” category for fearful examples reported lower difficulty and greater accuracy than those who selected non-matching emotion categories [difficulty: happy, *t*(4906) = −24.73, *P*<.001, fearful, *t*(4139) = −10.01, *P*<.001; accuracy: happy, *t*(4503) = 12.60, *P*<.001, fearful, *t*(3838) = 7.48, *P*<.001].

## Discussion

The recognition of human facial expressions, among the most fundamental aspects of emotion perception, has previously been shown to be sensitive to individual differences in social experience [3]–[8]. The results of the current study illustrate the role of experience as a modulator of interspecific emotion perception and thus extend the notion of experience-dependent processes in the development of this fundamental skill. Here, we find that in a large human cohort, individual differences in experience with dogs predict the perception of emotion in dogs.

Experience-associated effects were most pronounced in the interpretations of fearful, rather than happy, examples of dog behavior. For example, the likelihood of selecting the “fearful” category to describe fearful examples increased dramatically with experience, with the largest increase from the Low-Experience to the Owners group, while experience was not a significant predictor of selection of the “happy” category for happy examples. It is important to consider that the perception of happiness may not have varied with experience due to a ceiling effect. Since even the least-experienced respondents identified happiness in the depicted dogs at a high rate, there was little room for improvement. On the other hand, the lower rate at which dog owners, compared to professionals, identified fear suggests that professional experience with dogs aids proficiency in interpretations of fearful behavior. However, individuals who had been professionals for less than ten years did not vary significantly from more-experienced professionals in their categorizations of fearful or happy videos, indicating that interpretive skills may develop relatively early in a dog professional’s career.

Respondents’ self-ratings of difficulty and accuracy also varied with experience, with larger effects observed for interpretations of fearful than happy examples. For interpretations of fearful videos, difficulty ratings steadily decreased with experience until the professional level, while accuracy ratings increased. In contrast, for interpretations of happy videos, a decrease in difficulty ratings and increase in accuracy ratings was observed from the Low-Exp to the Owners group, with little additional change as experience increased. For all experience groups, lower difficulty and higher accuracy were reported for interpretations of happy than fearful examples. Since the self-ratings could be considered a measure of confidence in respondents’ own interpretive abilities, we can reasonably conclude that respondents were more confident when interpreting happy than fearful examples and that confidence grew with experience, particularly for interpretations of fearful behavior.

The discrepancies in results between interpretations of happy and fearful examples of dog behavior concur with intraspecific research. For example, studies on human facial expression recognition have consistently demonstrated that happy facial expressions are recognized at lower intensities and with higher accuracy and speed than fearful expressions [7], [19]–[21]. Furthermore, the ability to recognize happy expressions develops at an earlier age than the ability to recognize negative emotional expressions [22]–[25], and the perception of happy expressions is less influenced by individual differences in experiences, such as abuse [3]–[6].

The results of the current study are among the first to demonstrate that the perception of an emotion in dogs can be associated with human observers’ level of dog experience. Despite evidence that neural and visual activity in response to dog images varies according to experience with dogs [12], previous studies had found limited support for the effect of experience on the perception of emotion in dogs [9]–[11], [26]. While some studies on children found that the accurate decoding of emotion in dog vocalizations and facial expressions increased with age, it is unclear whether these effects were due to cumulative experience with dogs or the development of emotion processing systems [9], [10], [14], [26], [27]. In studies with adult participants, neither visual experience, nor experience with dogs, predicted accuracy in the interpretation of dog barks [9], [10], [26]. In addition, Tami and Gallagher [11] found only minimal effects of experience on interpretations of dog body language in dog-dog interactions. However, significant individual differences, even among dog professionals, in interpretations of dog-dog interactions may have contributed to their finding.

The effects of interspecific experience on interpretations of dogs’ visual signals in the current study, but not on vocalizations [9], [10], [26], could be explained by cross-species similarities in vocalizations. It has been suggested that mammals’ motivational and affective states are associated with acoustic qualities of their vocalizations, such as tonality and frequency [28]. For example, atonal, low-pitched vocalizations have been associated with aggression. Evidence for these basic structural-motivational rules of vocalizations has been identified in a variety of species, and these rules could enable the listener to accurately interpret vocalizations without extensive direct experience with a particular species. Although there are similarities in facial expressions across species [29], visual signals and their interpretation may display greater variation across species than vocalizations due to morphological differences in facial and bodily features. Therefore, direct experience with a species may be required in order to interpret its visual signals with a high degree of accuracy.

Humans are not the only species capable of acquiring another species’ signals through experience. For example, fathead minnows exposed to the chemical alarm cues of another species in the diet of a predator species subsequently display anti-predator responses to the alarm cues [30]. The superb fairy-wren, a species of passerine bird, acquires an ecologically sensitive recognition of alarm calls by noisy miner birds, only fleeing when hearing the calls in locations in which miners are present [31]. In these cases, individual animals that recognize heterospecific alarm cues likely benefit from increased fitness. It is also possible that accurate human recognition of other animals’ emotional signals may serve an evolutionarily relevant purpose, enabling the human observer to assess the danger posed by an animal and flee or approach accordingly.

### The Observation of Emotion in Dogs

A critical methodological feature of studies on the interspecific or intraspecific recognition of emotion is the identification of visual or auditory characteristics which are clearly associated with specific emotional states. There appears to be agreement among canid researchers and other dog professionals regarding the appearance of some emotional behaviors in dogs [32]–[35]. For example, fearful dogs are said to reduce their body size - crouching into a low posture, flattening their ears, and holding their tails in a low position [36], [37]. Shaking, yawning, salivation, freezing, panting, paw-lifting, and vocalizing are examples of other behaviors that have been associated with fear in dogs [38]–[41]. Such behaviors are also observed in dogs who have been diagnosed with fear-related disorders (e.g. thunderstorm phobia) [42]. These physical correlates to emotional states are reminiscent of Darwin’s “principle of antithesis” [29] whereby there are specific physical indices of an emotional state, and the opposite emotional state is accompanied by the opposite physical actions (*i.e.* slow, steady breathing while calm *vs.* panting when fearful; high tail position when confident *vs.* low tail position when fearful).

Accuracy in interpretations of emotional behaviors in dogs may be associated with observational patterns. For example, young children, who mistake aggressive dog faces as happy, tend to focus primarily on the mouth and teeth, rather than a scan of eyes, nose, and mouth [43]. Experience with dogs displaying a variety of emotions may facilitate the development of observational skills and increase the likelihood of focusing on species-appropriate features and behaviors. In the current study, the likelihood of reporting that facial features (eyes, ears, mouth/tongue) were emotionally informative increased from the non-professional to the professional level of experience, with the largest increase for the use of the ears. In contrast, differences by experience were smaller for selection of the bodily features (legs/paws, tail). Because of their relative size, bodily features may be salient targets of observation, even for less-experienced individuals. However, interestingly, participants who had never owned a dog were less likely than more-experienced individuals to report that the tail was informative when viewing both happy and fearful examples. The total number of features reported as emotionally informative also increased with experience up to the professional level, suggesting that a tendency to observe dogs more holistically grows with experience.

The results on observational techniques appear to contrast with earlier findings from eye-tracking studies. For example, Guo et al. found that non-owners gaze overwhelmingly more at the eyes than at the mouth in dogs’ faces [44]. In addition, Kujala et al. concluded that both experts and non-experts gaze more at dogs’ heads relative to dogs’ bodies [12]. Neither of these findings appears to be consistent with our results. However, the discrepancies could be due to methodological differences. First, while the eye-tracking studies measured actual duration of gaze on different parts of the dog, we asked participants to report on the parts of the dog that were emotionally informative. The two measures need not be correlated and represent distinct tasks (observation of a visual stimulus *vs.* interpretation of an emotional state). Second, photographs were used as stimuli in the eye-tracking studies, while videos were used in the current study. Third, neutral or unspecified emotion was depicted in the eye-tracking studies, while positive and negative emotions were included in this study. Intraspecific emotion recognition studies suggest that gaze varies based on the emotion observed. For example, humans use the eyes to decode fear in human facial expressions, while the mouth is used to decode happiness [45]. Therefore, gaze on dogs’ features may also vary based on the emotion displayed. While we did not track gaze, we found that facial features were more likely to be reported as informative in the interpretation of fearful than happy examples, while bodily features were more likely to be cited in interpretation of happy than fearful examples.

### Limitations of the Study

The use of stimulus sets in studies of both intraspecific and interspecific emotion perception are a potential methodological limitation as it may be the case that results are not generalizable to perceptions of emotion in live behavior. While an effort was made in the current study to include a diverse range of behaviors, breeds, and situations, there are aspects of live dog behavior, such as eye contact between dog and observer and contextual cues, which cannot be replicated in a video, and thus, it is possible that the effects of experience would be more modest in real-world observations.

Furthermore, since the stimulus set did not contain exhaustive examples of all fearful and happy behaviors, the results may not generalize to other examples of fear and happiness in dogs. We were also unable to explore the role of experience in interpretations of anger and sadness in dogs, since the expert panel did not identify examples of these emotions in the stimulus set with high agreement. It is possible that dog behavior experts do not agree on the appearance of anger or sadness in dogs, but it is also likely that the stimulus set simply did not contain clear examples of anger and sadness. In fact, relatively few examples of these emotions would be expected in typical, everyday scenarios. In studies of emotional experience, humans report feeling anger and sadness at a much lower rate than happiness [46], [47]. Similarly, one would not expect to find frequent examples of clear, high-intensity fear. Humans experience fear at least four times less frequently than happiness and at lower intensities [46], [47]. In addition, fear is generally more difficult to recognize than happiness, as previously noted [7], [19]–[21]. These factors may help explain why two of the fearful videos received lower agreement (75%) among the initial expert panel. Removing these videos did not change the patterns of results (see Text S1 for Supplementary Analyses).

Another limitation of the current study is that physiological measures were not included in the initial assessment of the dogs’ emotions. We should note, however, that the specific behaviors described by the initial expert panel in its assessment of fearful examples (*e.g.* lip-licking, panting, and low tail) have been linked to physiological indicators of stress and fear in dogs [39], [41]. Furthermore, the relationship between behavioral and physiological indicators of emotion is not always clear. For example, in a study of fear-conditioning in dogs, some dogs exhibited an increase in heart rate and body temperature, but no obvious behavioral changes [41]. A clinical study of thunderstorm-phobic dogs also demonstrated that cortisol levels, a physiological indicator of stress, were not associated with behavioral responses to thunder, such as panting, pacing, vocalization, and trembling [48]. In addition, cortisol levels were not associated with behavioral responses to social and spatial restriction and fear-inducing stimuli in some studies with shelter and laboratory dogs [38], [49]. Though the relationship between physiological and behavioral indicators of emotion is not always straightforward, incorporation of both types of measures could be informative in the future study of interspecific emotion perception.

Our methods did not account for potential differences among the experience groups in general social skills, including intraspecific emotion recognition. It is possible that differences in such social skills, rather than experience with dogs, could explain our results. Since many of the videos contained humans, the behavior of the humans could potentially have provided clues to viewers about the emotional states of the dogs. However, previous reports have found no differences between dog experts and non-experts in intraspecific empathy and perspective-taking [12]. Furthermore, if less-experienced respondents in the current study were indeed less adept at interpreting social cues in general, their identification of both fear and happiness in dogs should have been impaired, a pattern not supported by our data.

Lastly, the use of a convenience sample is a potential shortcoming of the current study. While the sample included a wide range of ages, more than 80% of the sample was female. The sex bias is not unusual for studies involving dog owners; previous studies on dog owners using questionnaire methodology have also reported that about 80% of respondents were female [50]–[52]. In addition, participants’ sex was taken into account in our analyses. However, it is possible that there were other unknown biases in the sample resulting from the use of “self-selected” individuals.

### Conclusion

Investigations of interspecific interpretations of behavior may promise to be an important new avenue for the study of fundamental social cognitive skills like emotion perception. In particular, they pose a unique opportunity to explore a broad range of experience in the development of such abilities. Until now, there has been limited evidence for the modulating effect of experience on the development of interspecific emotion perception [11], [13], even though intraspecific emotion perception has been known to be influenced by such processes [3]–[6]. Similarities between the findings of the current study and intraspecific emotion perception research suggest that the neural networks of emotion might be applied flexibly between interspecific and intraspecific contexts. In fact, it has recently been shown that children and adults who receive interspecific emotion recognition training not only improved their recognition of emotion in dogs, but also in humans [53].

On an applied level, our results also suggest that less-experienced individuals should be aware of potential deficits in their “dog-reading” ability when interacting with dogs. It has been suggested that misreading of dogs’ signals, particularly by young children, during interactions with dogs can lead to dog bites [54]. Education about dogs’ signals and appropriate dog-human interaction may ameliorate such miscommunication [55]–[58].

## Materials and Methods

### Stimulus Set

A panel of eight dog behavior professionals was recruited to characterize the emotional state of dogs displayed in 30 video clips. These individuals were recruited through recommendations from other behavior professionals, as well as personal contacts. The expert panel had a mean of 20 years of experience, and all had received certifications as pet behavior professionals. Two were diplomates of the American College of Veterinary Behaviorists (DACVB), two were certified applied animal behaviorists (CAAB), two were certified professional dog trainers (CPDT-KA), and two had received other certifications for dog behavior professionals.

The expert panel completed an online questionnaire, later also presented to participants. We focus our discussion on items within the scope of the current paper. For each video, the expert panel selected a single emotion category to describe the depicted dog (happy, sad, fearful, angry, neutral). The order of the emotion categories was randomized for each video. Experts provided a written description of the behaviors that helped them determine how each dog was feeling. Subsequently, 16 videos containing a diverse range of expert opinions were included in the stimulus set presented to the full sample of participants. These videos, each less than a minute long, were embedded in an online questionnaire and depicted dogs of various breeds and ages in a variety of everyday situations. The videos did not include sound due to the study’s focus on emotion perception using dogs’ visual signals. No more than two videos of a single breed were included, and a variety of behaviors and situations were depicted, except for dog-dog interactions. The videos were from a variety of sources, including professional videographers and dog behavior professionals. The videos were grouped into pairs with similar emotional content according to expert evaluations, and each participant received one video at random from each pair. Among the presented videos, there were nine videos on which at least six of eight experts (75%) had agreed in their emotion categorizations, and participants’ interpretations of these videos are the focus of the current analyses. According to this standard of expert agreement, five dogs were identified as happy, and four dogs were identified as fearful. Table 3 contains descriptions, as well as the level of expert agreement, for each of these videos. The supplementary materials contain a table listing technical details for each video (Table S1), as well as analyses excluding the two videos that received expert agreement of less than 100% (Text S1). An example of a video from the happy (Video S1–Video#9) and fearful (Video S2–Video#12) categories are included as supplementary files.

**Table 3:** Descriptions of Video Stimuli.

Video ID	Brief Description	Expert Categorizationand Percent Agreement	Expert Description of Specific Behaviors
1	Golden Retriever rolls on back in grass, looks around, and walks away.	Happy (100%)	Rolling calmly, loose tail wag, ears gently back, looking around with open mouth and relaxed commissures (corners of mouth), trots away with tail up and gentle wag
2	Border Collie sniffs ground, then approaches and greets woman.	Happy (100%)	Gentle tail wag, increasing during interaction; play bow; gentle jump onto person; relaxed eye contact; responsive to action of person; exploring environment without tensing muscles
3	Irish Setter stands on hind legs while licking man’s face.	Happy (100%)	Gentle mid-position tail wag, relaxed musculature, steady licking without turning away, not frenetic licking, responsive to handler’s movement
9	Maltese runs in the snow alongside a woman.	Happy (100%)	Running forward with upward bounce, tail up and loose, looking directly back at person with open mouth and brief eye contact, relaxed ears
10	Maltese walks around two people near a door and briefly jumps up on one of them.	Happy (100%)	Jumping up in a relaxed way; prancing around, face relaxed and loose; not avoiding petting; relaxed, high, flexible tail wag, voluntary climb onto person with muscles loose
5	Large mixed-breed is sitting indoors next to people and looks at camera from across the room.	Fearful (75%)	Very stiff, eyes wide open, ears forward, forehead wrinkled, mouth tight
6	Border Collie is held by standing woman, then placed on the ground.	Fearful (100%)	Squirming, stiff, licking lips, can see whites of eyes, facing away from person, attempts to escape once on ground
7	Medium mixed-breed barks at camera while moving from side to side behind handler.	Fearful (100%)	Stiff tail wagging, barking, jumping forward and then back, hiding behind person, holding ears and body back, slightly lowered tail while wagging in circular manner, not maintaining gaze
12	Shepherd mix stands just outside a screen door, facing the camera.	Fearful (75%)	Low and fast tail wag, tense areas around dog’s eyes and muzzle, heavy panting, head-turns away from camera, ears pressed back, weight slightly shifted to back end, huge tongue, eyes bulging

Brief descriptions of the scenario displayed in each video, emotion categorizations with percent agreement in initial expert panel, and examples of detailed behavioral descriptions provided by experts. Supplementary analyses (Text S1), excluding the two videos (5 and 12) that received lesser agreement, produced very similar findings as those reported in the *Results*.

### Recruitment

A recruitment website was created that briefly described the research and contained a link to the questionnaire, which could be completed and submitted online. Participants were recruited through a variety of means, including online postings, e-mails, press releases, flyer distribution at dog events, and an undergraduate participant pool. All procedures were approved by the Columbia University Institutional Review Board.

### Questionnaire

After consenting to participate in the study, respondents provided demographic information about themselves, as well as information about their experience with dogs. They could indicate whether they had worked professionally with dogs, owned a dog at any point in their lives, or never or only occasionally interacted with dogs. In addition, if they had worked with dogs professionally, they were asked to provide the number of years of professional interaction, as well as the type of professional work (e.g. dog behavior professional, veterinarian, dog walker, dog groomer). Participants also indicated if and how they had learned to interpret the body language of dogs (e.g. by reading a book or article, attending a lecture, watching a video).

After viewing each video, respondents were provided with the same emotional interpretation questions presented to the initial expert panel. First, they were asked to categorize the emotion displayed by the dog (angry, fearful, happy, neutral, sad). Respondents also indicated which specific features of the dog helped them determine how the dog was feeling (eyes, ears, mouth/tongue, legs/paws, tail). Multiple selections were permitted. In addition, respondents rated on a nine-point scale how difficult it was to determine how each dog was feeling (1 = very easy, 9 = very difficult) and how accurate they believed their responses to be (1 = very inaccurate, 9 = very accurate).

The final question of the survey asked respondents to rate on a nine-point scale how likely or unlikely it is that dogs experience each of the following emotions (1 = very unlikely, 9 = very likely): happiness, anger, sadness, fear, guilt, surprise, love, frustration, excitement, and disgust. Likeliness ratings for happiness and fear were used in the analyses of emotion categorizations.

### Statistical Analyses

Responses to happy and fearful examples were analyzed separately for all measures. Logistic regression with generalized estimating equations was used to explore the relationship between participants’ level of experience with dogs and their categorizations of emotion in the viewed dogs [59]. Generalized linear models were performed using the GENLIN program in SPSS 17.0. For the dogs assessed by the initial expert panel as happy, participants’ selection of the “happy” category was coded as “1,” while selection of other emotion categories was coded as “0.” For the fearful examples, selection of the fearful category was coded as “1,” while selection of other categories was coded as “0.” For these and subsequent analyses, the videos were entered as a repeated measure for each participant, and the experience variable was entered as a fixed effect. In addition, participant’s sex, participant’s age, and a variable containing video identification numbers were included in each model in order to account for the effects of sex, age, and individual videos. The *Wald X^2^* statistic, which is similar to the *t*-statistic in linear regression, is presented in the results, and where significant, indicates that the experience variable was a significant predictor of emotion categorization, even after adjusting for the other variables in the model. Pairwise comparisons were conducted among the experience groups, and *p*-values were Sidak-corrected for multiple comparisons.

Another model was conducted that also controlled for respondents’ ratings of the likeliness that dogs experience happiness or fear. For example, a variable containing respondents’ ratings of the likeliness that dogs experience fear was added as a covariate in order to determine the effect of experience on fearful categorizations after accounting for perceptions of the likeliness of fear in dogs. Lastly, in order to focus on the effect of hands-on experience, rather than knowledge acquired through other means, on interpretations of dogs’ visual signals, an additional analysis was performed on a subset of respondents who reported that they had never learned about dog body language by reading a book or article, watching a video, attending a lecture, or receiving an explanation from a behavior professional. For this analysis, responses from the two professional groups were combined due to smaller group sizes.

Poisson regression with generalized estimating equations was conducted on the number of features that respondents reported as emotionally informative. In addition, logistic regression with generalized estimating equations was conducted on selection of each feature. Separate models were run on each of the five body part categories with responses coded as “0” (non-selection) or “1” (selection). Video 5 was excluded from analyses of observational focus, since parts of the dog were not visible (Table S1). Lastly, difficulty and accuracy ratings were analyzed with linear mixed models. For all of these analyses, the videos were entered as a repeated measure for each participant, and the experience variable was entered as a fixed effect, along with participant’s sex, participant’s age, and the video identification variable. Additional models were performed on difficulty and accuracy ratings for fearful and happy examples that included as a predictor variable the matching or non-matching status of participant and expert categorizations.

## Supporting Information

Table S1 **Additional information regarding video stimuli.**
(DOCX)

Text S1 **Supplementary analyses.**
(DOCX)

Video S1 **Video of “happy” emotional example (Video#9).**
(MP4)

Video S2 **Video of “fearful” emotional example (Video#12).**
(MP4)
