# Supplementary material for: Human Perception of Fear in Dogs Varies According to Experience with Dogs
Source: PLoS One. 2012 Dec 19;7(12):e51775. doi: 10.1371/journal.pone.0051775 (PMC3526646; doi:10.1371/journal.pone.0051775)
Supplement: Table S1 — Additional information regarding video stimuli. (DOCX) [file pone.0051775.s001.docx]

**Table S1. Additional Information Regarding Video Stimuli.**

| **Video ID** | **Expert Categorization** | **Length of Video** | **Dimensions of Video (pixels)** | **Whole Dog Displayed** | **Size of Dog Relative to Background** | **Human Present** |
| --- | --- | --- | --- | --- | --- | --- |
| 1 | Happy | 15 s | 640 X 480 | Yes | 25-50% | No |
| 2 | Happy | 10 s | 640 X 480 | Yes | < 25% | Yes |
| 3 | Happy | 6 s | 640 X 480 | Yes | < 25% | Yes |
| 9 | Happy | 5 s | 640 X 480 | Yes | < 25% | Yes |
| 10 | Happy | 15 s | 640 X 480 | Yes | < 25% | Yes |
| 5 | Fearful | 3 s | 640 X 480 | No (Tail not visible, legs partially visible.) | 25-50% | Yes |
| 6 | Fearful | 12 s | 640 X 480 | Yes | < 25% | Yes |
| 7 | Fearful | 31 s | 640 X 480 | Yes | < 25% | Yes |
| 12 | Fearful | 18 s | 640 X 480 | Yes | < 25% | No |

Emotion categorizations by initial expert panel, along with characteristics of each video. Video 5 was removed from analyses of reported observational focus, since the whole dog was not displayed. Supplementary analyses (Text S1) exclude Videos 5 and 12.
