# Supplementary material for: Human Perception of Fear in Dogs Varies According to Experience with Dogs
Source: PLoS One. 2012 Dec 19;7(12):e51775. doi: 10.1371/journal.pone.0051775 (PMC3526646; doi:10.1371/journal.pone.0051775)
Supplement: Text S1 — Supplementary analyses. (DOCX) [file pone.0051775.s002.docx]

**S1. Supplementary Analyses.**

The two fearful videos (5 and 12) that received expert agreement below 100% were excluded in the following analyses (Video 5 had already been excluded in analyses of observational techniques that were presented in the *Results*, since the entire dog was not visible). Unless otherwise noted, the findings presented below do not vary from those reported in the *Results*.

Emotion Categorizations: Experience remained a significant predictor of selection of the “fearful” category to describe the fearful examples, *Wald* *X^2^*(3, *N* = 1648) = 53.19, *P* < .001. Sig. pairwise comparisons (Sidak-corrected): Low-Exp < Own < Prof<10 = Prof10+.

Number of Dog’s Features: Experience remained a significant predictor of the number of features that participants reported as emotionally informative in fearful exmaples, *Wald* *X^2^*(3, *N* = 1648) = 112.86, *P* < .001. Sig. pairwise comparisons (Sidak-corrected): Low-Exp < Own < Prof<10 = Prof10+.

Eyes: Experience remained a significant predictor of reporting of the eyes as emotionally informative in the fearful examples, *Wald* *X^2^*(3, *N* = 1648) = 62.48, *P* < .001. Sig. pairwise comparisons (Sidak-corrected): Low-Exp = Own < Prof<10 = Prof10+.

Ears: Experience remained a significant predictor of reporting of the ears as emotionally informative in the fearful examples, *Wald* *X^2^*(3, *N* = 1648) = 96.15, *P* < .001. Sig. pairwise comparisons (Sidak-corrected): Low-Exp < Own < Prof<10 = Prof10+.

Mouth/Tongue: Experience remained a significant predictor of reporting of the mouth as emotionally informative in the fearful examples, *Wald* *X^2^*(3, *N* = 1648) = 70.93, *P* < .001. Sig. pairwise comparisons (Sidak-corrected): Low-Exp = Own < Prof<10 = Prof10+.

Legs/Paws: Experience was a significant predictor of reporting of the legs as emotionally informative in the fearful examples, *Wald* *X^2^*(3, *N* = 1648) = 11.11, *P* = .01. Sig. pairwise comparisons (Sidak-corrected): Prof<10 = Own < Low-Exp. (In previously reported results, this finding was not significant)

Tail: Experience remained a significant predictor of reporting of the tail as emotionally informative in the fearful examples, *Wald* *X^2^*(3, *N* = 1648) = 18.37, *P* < .001. Sig. pairwise comparisons (Sidak-corrected): Low-Exp < Own = Prof<10 = Prof10+.

Difficulty: Experience remained a significant predictor of difficulty ratings for fearful videos, *F*(3, 2180) = 22.88, *P* < .001. Sig. pairwise comparisons (Sidak-corrected): Prof10+ = Prof<10 < Own = Low-Exp. (In previously reported results, Own < Low-Exp)

Accuracy: Experience remained a significant predictor of accuracy ratings for fearful videos, *F*(3, 2162) = 19.73, *P* < .001. Sig. pairwise comparisons (Sidak-corrected): Low-Exp < Own < Prof<10 = Prof10+.
